# A Fork in the Road for Emergency Medicine and Critical Care Blogs and Podcasts: Cross-sectional Study

**DOI:** 10.2196/39946

**Published:** 2022-10-28

**Authors:** Michelle Lin, Mina Phipps, Yusuf Yilmaz, Christopher J Nash, Michael A Gisondi, Teresa M Chan

**Affiliations:** 1 Department of Emergency Medicine University of California San Francisco San Francisco, CA United States; 2 Department of Emergency Medicine Stanford University Stanford, CA United States; 3 Education Research, Innovation, and Theory Program McMaster University Hamilton, ON Canada; 4 Department of Medical Education Faculty of Medicine Ege University Izmir Turkey; 5 Department of Emergency Medicine Massachusetts General Hospital Boston, MA United States; 6 Precision Education and Assessment Research Lab Department of Emergency Medicine Stanford University Stanford, CA United States; 7 Division of Emergency Medicine Department of Medicine McMaster University Hamilton, ON Canada

**Keywords:** open educational resource, free open-access meducation, FOAM, meducation, open-access, internet based, web based, website, social media, medical education, disruptive innovation, blog, podcast, emergency, critical care

## Abstract

**Background:**

Free open-access meducation (FOAM) refers to open-access, web-based learning resources in medicine. It includes all formats of digital products, including blogs and podcasts. The number of FOAM blog and podcast sites in emergency medicine and critical care increased dramatically from 2002 to 2013, and physicians began to rely on the availability of these resources. The current landscape of these FOAM sites is unknown.

**Objective:**

This study aims to (1) estimate the current number of active, open-access blogs and podcasts in emergency medicine and critical care and (2) describe observed and anticipated trends in the FOAM movement using the Theory of Disruptive Innovation by Christensen as a theoretical framework.

**Methods:**

The authors used multiple resources and sampling strategies to identify active, open-access blogs and podcasts between April 25, 2022, and May 8, 2022, and classified these websites as blogs, podcasts, or blogs+podcasts. For each category, they reported the following outcome measures using descriptive statistics: age, funding, affiliations, and team composition. Based on these findings, the authors projected trends in the number of active sites using a positivist paradigm and the Theory of Disruptive Innovation as a theoretical framework.

**Results:**

The authors identified 109 emergency medicine and critical care websites, which comprised 45.9% (n=50) blogs, 22.9% (n=25) podcasts, and 31.2% (n=34) blogs+podcasts. Ages ranged from 0 to 18 years; 27.5% (n=30) sold products, 18.3% (n=20) used advertisements, 44.0% (n=48) had institutional funding, and 27.5% (n=30) had no affiliation or external funding sources. Team sizes ranged from 1 (n=26, 23.9%) to ≥5 (n=60, 55%) individuals.

**Conclusions:**

There was a sharp decline in the number of emergency medicine and critical care blogs and podcasts in the last decade, dropping 40.4% since 2013. The initial growth of FOAM and its subsequent downturn align with principles in the Theory of Disruptive Innovation by Christensen. These findings have important implications for the field of medical education.

## Introduction

Many health care professionals abandoned their use of traditional medical textbooks over the last decade in favor of open-access, web-based, digital learning resources [1-5]. These resources and the trends to use them are collectively termed FOAM, for free open-access meducation [6,7]. We define FOAM as medical education resources produced in any format that are freely available online and across many different platforms without a required log-in. Examples include medical blogs, podcasts, subscription-free websites, and crowdsourced materials that constantly evolve. As the popularity of FOAM resources grew, so did the number and quality of these products [6]. In 2014, Cadogan et al [8] reported that 183 free blogs and podcasts were active in the fields of emergency medicine and critical care alone. This represented an approximately 90-fold increase in prevalence of these learning resources over the previous 10-year period.

The FOAM movement democratized and disrupted medical education by providing rapid, convenient, and open access to a breadth of clinical content on demand [9,10]. Globally, providers in practice and trainees both use FOAM for personal learning and in formal health professions curricula [11-13]. However, recent evidence shows that trainees seem to *primarily* use trusted blogs and podcasts when building their foundational medical knowledge [5,14]. Especially with the COVID-19 pandemic driving education toward more web-based content, the integration of FOAM in formal curricula has become more commonplace [4,15].

It is unclear if the current landscape of open-access blogs and podcasts can meet the demand and needs of modern learners [16]. The *Theory of Disruptive Innovation* by Christensen provides a conceptual framework to map the growth of these resources, anticipate roadblocks, and forecast new developments [17,18]. Christensen suggests that innovation by smaller entities (disruptors) in a market can challenge and may displace established organizations (incumbents). Incumbents may initially overlook disruptors because they initially seem trivial, underresourced, or too niche to succeed. Disruptive innovations typically start as either low-end or new-market footholds, catering to a low-cost idea or a new segment of an existing market to reach underserved customers. Over time, when the disruptor crosses a mainstream acceptability threshold, it begins to overtake all or part of the incumbent’s market share, and eventually profitability, because it is less expensive or more accessible.

In this framework, FOAM is a market-creating, disruptive type of innovation in medical education [19-21]. New-market innovations seek to fulfill a need in an unserved population—sometimes to provide goods or services offered by incumbent entities to those populations that could not afford or could not access those products. Although the new-market innovation targets a nonconsumption market (providing a good or service that was previously nonexistent to its audience), it still directly challenges the incumbent’s market space by potentially attracting away some of the incumbent’s customers with its new, affordable, or accessible option. The FOAM movement followed this new-market disruption model as free blogs and podcasts grew to establish a foothold in the market of knowledge translation and information dissemination. They were created in response to the incumbents (print textbooks, journals, live conferences, and professional societies) to aid health care providers and trainees who previously could not have easily accessed paywalled literature [12,22]. Prior to the FOAM movement, clinicians would access information by reading the original research from journal articles, attending conferences, or reading subscription-based references. Emergency medicine and critical care blogs and podcasts entered the market in 2002, and continued improvement cycles by bloggers and podcasters made these open-access resources more appealing and acceptable for learning and clinical practice. This was evidenced in 2002-2013 when there was an exponential growth in the number of blogs and podcasts in emergency medicine and critical care. Although FOAM would likely never entirely replace incumbent resources [7], it still attracted many end users and thus siphoned market share.

As medical education continues to evolve, one might anticipate that open-access blogs and podcasts would at some point supplant incumbent sources of content. However, many of the blogs and podcasts identified in 2014 by Cadogan et al [8] were small in scale and relied on the volunteerism efforts and financial resources of dedicated individuals [6]. Which of these blogs and podcasts remain active today is unknown, and that gap has far-reaching implications for medical education and knowledge translation. Therefore, this study aims to (1) estimate the current number of active, open-access blogs and podcasts in emergency medicine and critical care and (2) describe observed and anticipated trends in the FOAM movement using the *Theory of Disruptive Innovatio*n by Christensen as our theoretical framework [17,18].

## Methods

### Study Design

Using a positivist paradigm (a form of hypothesis testing through observation and measurements to build explanatory associations [23]) and *Theory of Disruptive Innovation* by Christensen as a theoretical framework [17,18], we conducted a cross-sectional study that identified active, open-access emergency medicine and critical care blogs and podcasts.

### Data Collection

We identified active websites between April 25, 2022, and May 25, 2022, using a similar methodological approach as that described in 2014 by Cadogan et al [8]. More specifically, we created a roster of blogs and podcasts by combining a list created by the *Life in the Fast Lane* organization in 2019 with *Feedspot’s* “80 Best Emergency Medicine Blogs and Websites” list in 2022 [24]. Subsequently, we identified additional sites through a purposeful snowball sampling technique, personal communications, social media, and a self-report form published as a blog post solicitation on the *Academic Life in Emergency Medicine* website on May 4, 2022. Lastly, we performed a Google search during May 1-8, 2022, to identify any overlooked sites, using a Boolean search strategy with the following terms: (“emergency medicine” OR “critical care” OR “intensive care”) AND (“podcast” OR “blog”).

### Inclusion and Exclusion Criteria

We included emergency medicine and critical care websites if they published at least one post in the previous 6 months, were free and open-access, were composed in English, did not require a log-in or subscription, served health care professionals as the audience, and published content intermittently in dated posts. We then classified these websites into *blogs* (content was primarily text), *podcasts* (content was primarily audio, which may include text-based show notes), or *blogs+podcasts*. We excluded websites if they covered a broad range of specialties, of which emergency medicine or critical care comprised the minority of topics. Two study investigators (ML and MP) adjudicated uncertainties regarding website inclusion and classifications.

### Outcome Measures

We collected the following data for each website: date of first published post, commercial ads used on the home page, other funding strategies (such as sales of books, web-based courses, or merchandise), institutional affiliation (sponsored by a professional society, journal, or external nonprofit or for-profit organization), and the personnel composition of the website (number of administrators, professional identity, and clinical practice setting affiliation).

### Statistical Analysis

We used descriptive statistics to summarize our results. We tallied the total number of active emergency medicine and critical care websites and noted the number of blogs, podcasts, and blogs+podcasts. For each of these 3 categories, we report the median age of the website and each outcome measure.

### Ethical Considerations

This study does not involve human participants. Therefore, we did not require institutional review board approval for this study because the information was publicly available.

## Results

As of May 2022, we report 109 emergency medicine and critical care websites, comprised of 50 (45.9%) blogs, 25 (22.9%) podcasts, and 34 (31.2%) blogs+podcasts, as well as their characteristics (Table 1). The educational sources ranged in age from less than a year to nearly 18 years. Sponsorship of these sites varied, including advertisements (n=20, 18.3%) and institutional sources (n=48, 44%). Many sites (n=30, 27.5%) used nontraditional sources of revenue, such as the selling of merchandise, books, web-based courses, and premium podcast content. For some sites (n=30, 27.5%), a funding source was not immediately apparent. Team sizes ranged from single-person authors to large teams of at least 5 individuals, with most (n=60, 55%) comprised of at least 5 people. Physicians led most sites (n=97, 88.1%).

**Table 1:** Characteristics of active, open-access emergency medicine and critical care websites in 2022.

Characteristics					Blogs (n=50)			Podcasts (n=25)			Blogs+podcasts (n=34)			All sites (n=109)
Age in years, median (range)					8.3 (0.8-17.7)			4.3 (1-10.6)			8.8 (4.6-15.3)			7.1 (0.8-17.7)
**Sponsorship, n (%)**														
	Advertisements			8 (16.0)			2 (8.0)			10 (29.4)			20 (18.3)	
	Other funding strategies			13 (26.0)			3 (12.0)			14 (41.2)			30 (27.5)	
	Institutional			21 (42.0)			14 (56.0)			13 (38.2)			48 (44.0)	
	No ads, funding strategies, or institutional affiliation			16 (32.0)			8 (32.0)			6 (17.6)			30 (27.5)	
**Team composition**														
	**Number of individuals, n (%)**													
		1	11 (22.0)			7 (28.0)			8 (23.5)			26 (23.9)		
		2-4	8 (16)			10 (40)			4 (11.8)			22 (20.2)		
		≥5	30 (60.0)			8 (32)			22 (64.7)			60 (55.0)		
		Unknown	1 (2.0)			0 (0)			0 (0)			1 (0.9)		
	≥1 member in academia			34 (68.0)			20 (80.0)			24 (70.6)			78 (71.6)	
	Physician-led			45 (90.0)			21 (84.0)			30 (88.2)			97 (88.1)	

## Discussion

### Principal Findings

**Figure 1:**
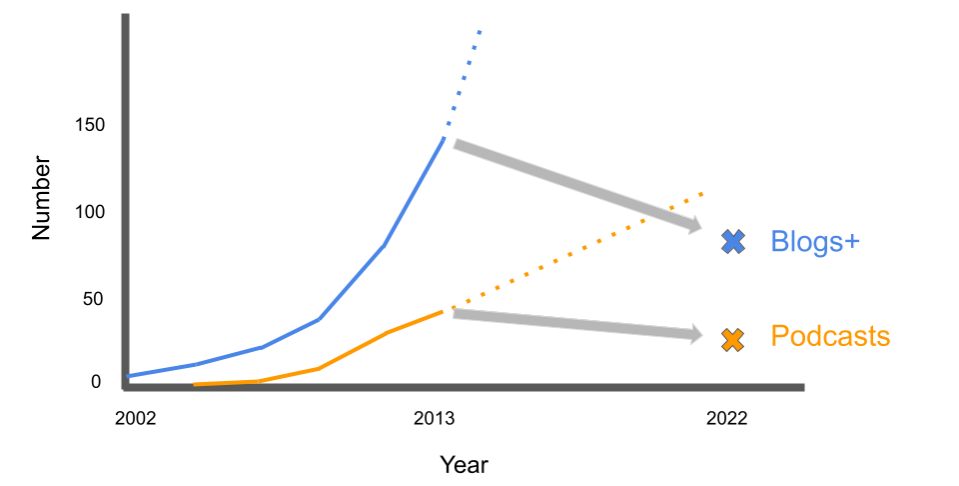
Number of active blogs+ (defined as blogs or blogs with podcasts) and podcasts during 2002-2013 compared to 2022. The dotted lines represent predicted trends if growth had continued at a consistent rate.

The attrition rate of FOAM websites should alarm medical educators. We found that the total number of free blogs and podcasts in emergency medicine and critical care plummeted by 40.4% when compared to the 2014 report by Cadogan et al [8]. Figure 1 depicts the trends of FOAM blogs and podcasts over time, highlighting the deviation of the present-day landscape from extrapolations based on previous trends. This decline in FOAM is an unforeseen shift in the medical education marketplace that is particularly concerning given the vast number of learners who depend on the stable availability of these resources.

The Christensen *Theory of Disruptive Innovation* helps us understand why fewer FOAM sites currently exist [18]. We posit that incumbents, such as professional societies and medical journals, launched their own educational blogs and podcasts, leveraging existing administrative staff and resources unavailable to the individual blogger or podcaster. This hybridization strategy by incumbents follows the disruptive innovation theory, which states that when the incumbent entity recognizes that a disruption is encroaching on its market space (and profitability), co-opting the disruptive innovation can retain market dominance and relevance [25]. Such incumbent activity may have lessened the need and urgency for new sites and thus reduced the scale of any continued market disruption by those smaller entities.

### Financial Sustainability

Sustained success requires a foundation of infrastructure and resources. The disruptive innovation framework confirms this financial reality and explains why existing FOAM sites may be dissolving. The framework’s premise is that successful disruptors eventually become more financially solvent as they encroach on the incumbents’ market and customers. The FOAM movement, however, is grounded in a social good mission by volunteers providing free education to all who wish to learn. This volunteerism comes at the expense of opportunity costs and may have been unsustainable for many sites that no longer exist. Some sites likely had to increase their team size with more authors, editors, and administrators willing to donate their time and efforts. This is demonstrated by our finding that most sites now have at least 5 team members. Interestingly, fewer open-access FOAM sites may have resulted from previously free blogs or podcasts opting into a paid subscription business model to help maintain financial stability. Indeed, the increasing financial pressures on FOAM producers have caused some to shift their business model entirely to ensure the viability of their outlets. Our analysis found that a substantial number of FOAM sites revised their business plans. Of the 109 active sites, only about one-quarter (n=30, 27.5%) do not generate funds from advertisements, sales, or sponsorship. More specifically, 18% (n=20) embed ads; 27.5% (n=30) use a sales model that asks for donations or payment for merchandise, continuing medical education credits, books, or web-based courses; and 44% (n=48) are affiliated with a sponsoring institution, such as a professional society, hospital, or journal.

### Academic Sustainability

Similar to financial opportunity costs, there are also academic opportunity costs. Traditionally, academicians are promoted and rewarded for their scholarly efforts based on grant funding, peer-reviewed publications, and national reputations. Digital scholarship in the form of blogs and podcasts has less academic value and is therefore less beneficial to career promotions and advancement [26,27]. This may play a role in the diminishing contributions of academicians, who play roles in 71.6% of existing FOAM sites, in favor of endeavors of higher academic value. Academic benefit, however, does not explain the decline in FOAM sites created by educators not based in academia.

### Higher Expectations

FOAM consumers have become accustomed to well-designed websites, rigorously produced content, up-to-date recommendations, and means for curricular integration of resources, and their expectations continue to rise. The volunteer-driven enthusiasm of many sites may have waned because of these production pressures placed upon FOAM producers over time. As another disincentive toward creating one’s own FOAM site, several existing sites allow guest authors to publish on their platform, providing educators with an outlet to disseminate their work with less investment of their time and resources. Consequently, the FOAM movement may be a victim of its own success—as the resources used most frequently by health care professionals and trainees matured, there was a natural imperative for some sites to grow or close. Our findings suggest a volatile educational marketplace with resources coming and going, threatening the reliance on and longitudinal use of these resources by stakeholders.

**Figure 2:**
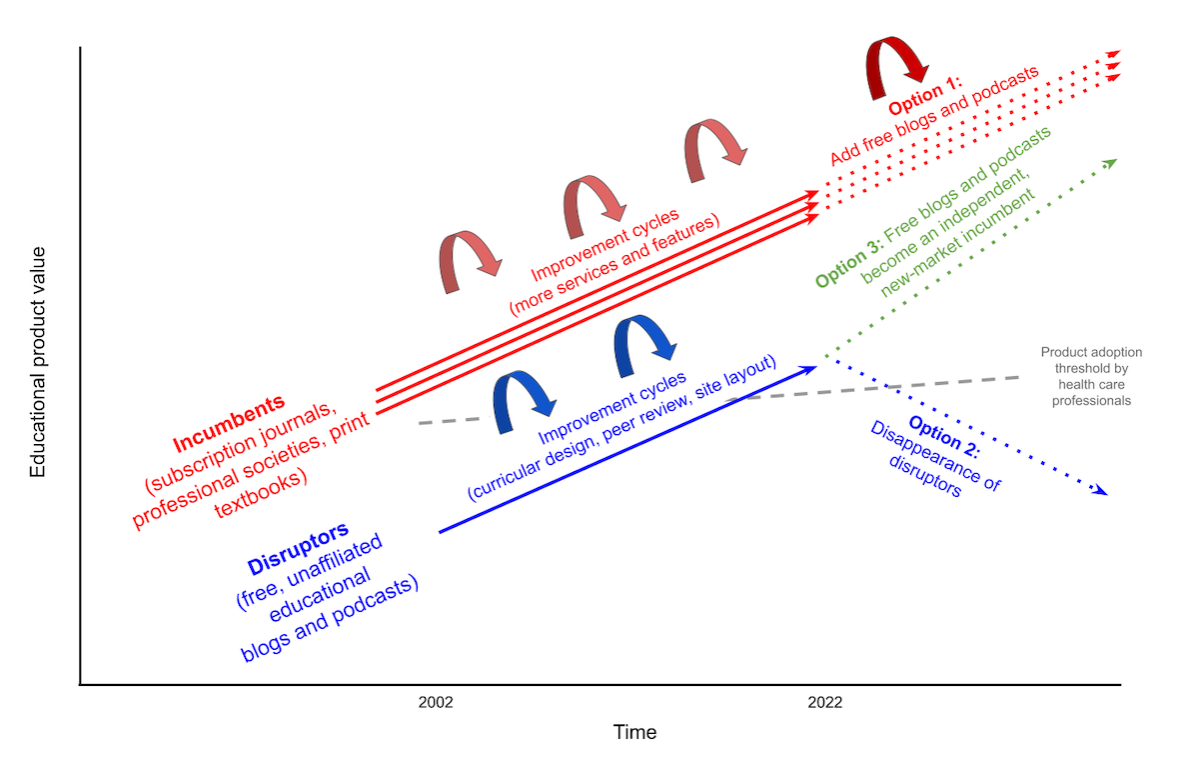
Disruptive innovation model of free educational blogs and podcasts for knowledge translation, adapted from the Christensen Theory of Disruptive Innovation, illustrating its three potential futures for the FOAM (free open-access meducation) movement: joining existing incumbents as a sustaining innovation (option 1), disappearing (option 2), or remaining nonpartisan and sustainable as a new-market incumbent (option 3). The gray dashed line signifies the rising threshold of product quality by the audience before they will use it.

Taken altogether, this turnover in enduring FOAM sites is concerning for trainees and educators who currently rely on the stability of blogs and podcasts for planned learning and curriculum use. Looking forward, the reliance on the volunteerism and the financial investments of a few individuals seems not to be a sustainable model for the FOAM movement. It thus seems fated to follow one of two paths, based on our adapted version of the Christensen construct (Figure 2). One is to ultimately be co-opted by organizations with the financial means and infrastructure to support ongoing content production, such as journals, publishers, professional societies, academic institutions, and for-profit organizations (option 1). The FOAM movement will still technically exist but will instead report to a partisan parent organization. Examples include the following: *EM News*, which hosts a blog under its publisher Wolters Kluwer; the podcast *Annals of Emergency Medicine*, which highlights its own journal findings; the podcast *EM Cases*, which is sponsored by the not-for-profit organization Schwartz/Reisman Emergency Medicine Institute; the blog and podcast platform of *Core EM*, which is hosted by the NYU/Bellevue emergency medicine residency program; and the podcast *EM Over Easy*, which serves as the official podcast for the American College of Osteopathic Emergency Medicine. The other path is that FOAM sites dissolve over time (option 2). While some attrition or business remodeling has occurred, we still have not seen most blogs and podcasts choose a path one way or the other. We believe the next several years will be crucial as most sites will commit themselves to a particular path. One can envision a third theoretical path (option 3) resulting in a new-market model of an independent FOAM site with continued open-access content and alternative revenue streams to maintain financial stability.

### The Future of FOAM

At its core, the FOAM movement is a social-good, disruptive innovation that provides high-quality, open-access educational blogs and podcasts for health care professionals. The FOAM movement has made significant inroads into the medical education and digital scholarship landscape. For instance, some sites have incorporated learning management platforms [28,29], curricular lesson plans for trainees [30-32], virtual communities of practice [33-37], and digital object identifiers for blog posts or podcasts, making them eligible for Altmetric Attention Scores [38]. However, without more infrastructure and added financial and academic security, the FOAM movement seems destined for either assimilation into traditional incumbent institutions or disbandment.

We predict that if the FOAM sites are to achieve independence and sustainability (Figure 2, option 3), the solution will rest upon finding successful business models. The web-based Khan Academy may serve as such a model. As a nonprofit organization, it provides open-access educational videos and a learning management system for any educator or learner to use for free as an adjunct to traditional classroom teaching. It remains operational through philanthropic donations and sales of niche educational services. By contrast, however, the FOAM movement is a fluid, decentralized, virtual community. If FOAM similarly adopts a philanthropic means for sustainability, we anticipate that funders will likely gravitate toward a limited number of the highest-quality or the most impactful blogs and podcasts to start. This future would require a means to objectively measure the quality of these sites in an equitable and transparent manner, such as with the Social Media Index [39,40].

We will monitor the availability of open-access blogs and podcasts in emergency medicine and critical care to understand whether the observed trends from our study continue or worsen. The aim is to detect early unexpected signals of change regarding growth, content quality, audience reach, sustainability, and impact.

### Limitations

Several important limitations to this study must be mentioned. We acknowledge that we may have overlooked some active blogs or podcasts, though we believe our search strategy was rigorous. Moreover, we may have erroneously included or excluded identified sites from our final list. However, we feel that small errors in identifying sites would not have significantly affected our core finding of a downward trend in FOAM. Another limitation involves generalizability of our findings because we only studied emergency medicine and critical care sites. Other medical specialties may have developed more sustainable models, but we identified none reported in the literature. The COVID-19 pandemic also is an outlier that may have affected the FOAM movement from 2020 to 2022, though we believe that there were likely more users of web-based content at that time, not fewer.

### Conclusion

The exponential growth of FOAM blogs and podcasts in the fields of emergency medicine and critical care has taken a sharp downturn. The new-market creation, initial growth, and attrition patterns of these resources align with the Christensen *Theory of Disruptive Innovation*. The FOAM movement is at a fork in the road with three potential futures, which are assimilation by traditional entities, continued attrition, or new financial sustainability. Our findings have important implications for learners, educators, and the field of medical education.

## Abbreviations

ALiEM: Academic Life in Emergency Medicine
FOAM: free open-access meducation
